# Supplementary figures and images for: UHPLC-ESI-QE-Orbitrap-MS based metabolomics reveals the antioxidant mechanism of icaritin on mice with cerebral ischemic reperfusion
Source: PeerJ. 2023 Jan 10;11:e14483. doi: 10.7717/peerj.14483 (PMC9838208; doi:10.7717/peerj.14483)

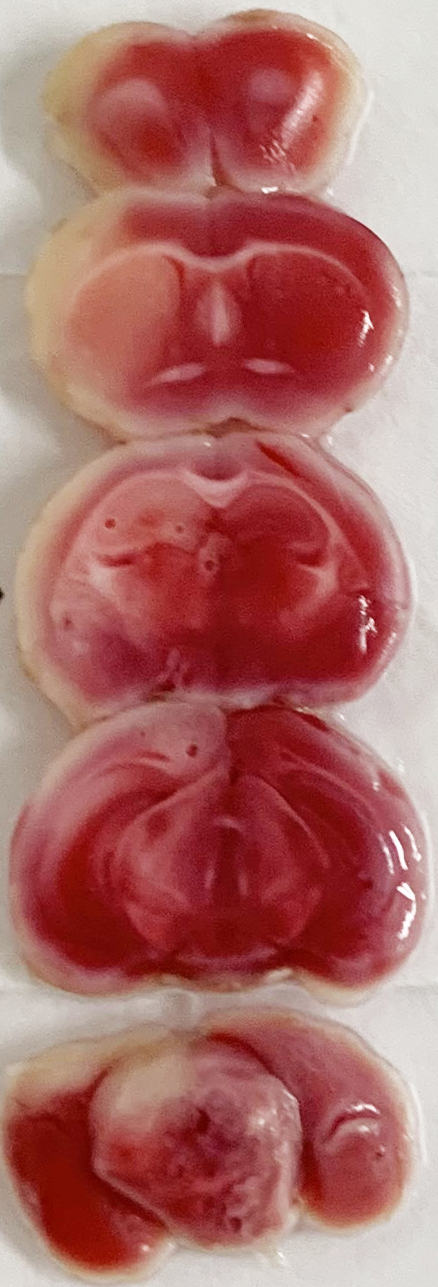

Supplement: Supplemental Information 1 [file peerj-11-14483-s001.zip › Original figures for cerebral infarction/ICT/ICT1.jpg]

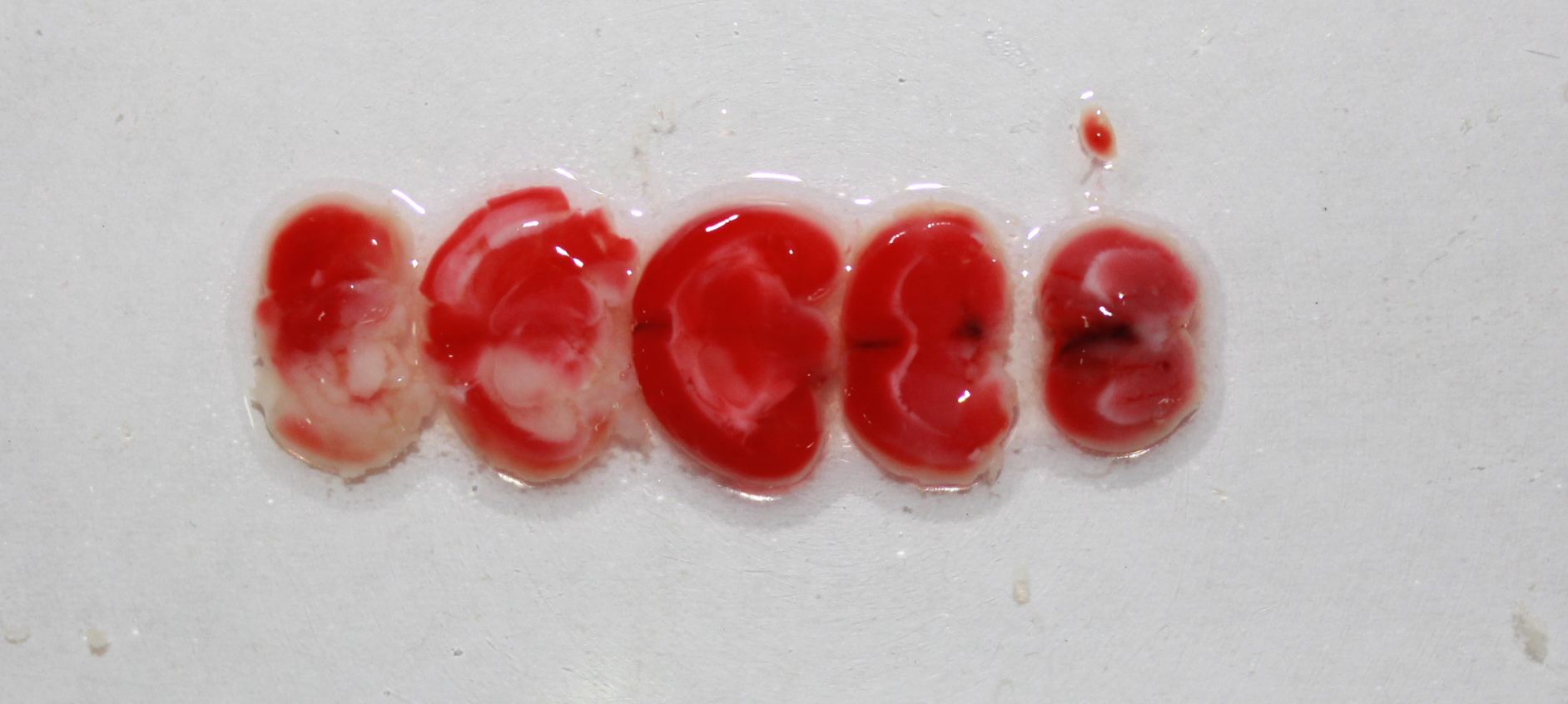

Supplement: Supplemental Information 1 [file peerj-11-14483-s001.zip › Original figures for cerebral infarction/ICT/ICT2.jpg]

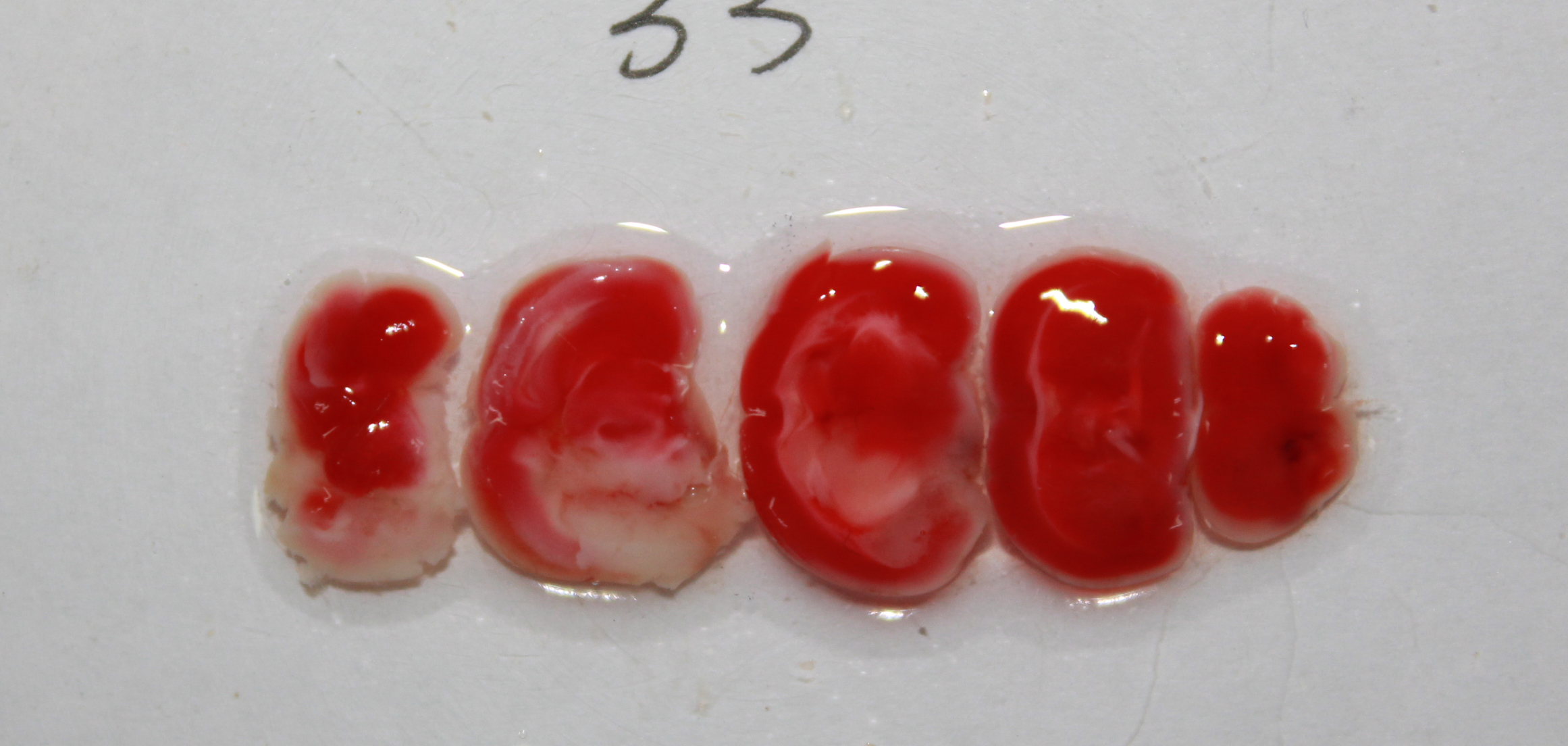

Supplement: Supplemental Information 1 [file peerj-11-14483-s001.zip › Original figures for cerebral infarction/ICT/ICT3.jpg]

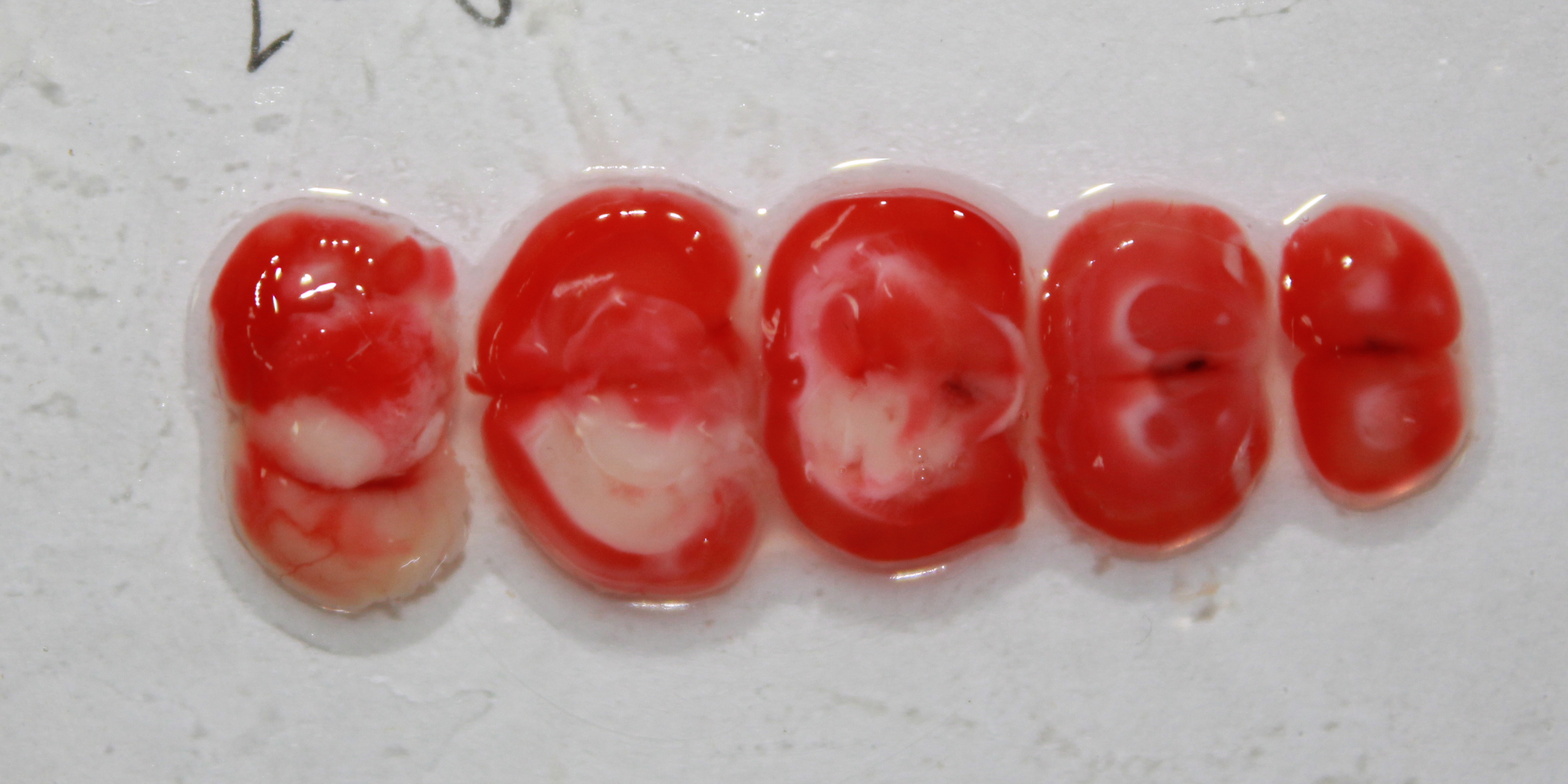

Supplement: Supplemental Information 1 [file peerj-11-14483-s001.zip › Original figures for cerebral infarction/ICT/ICT4.jpg]

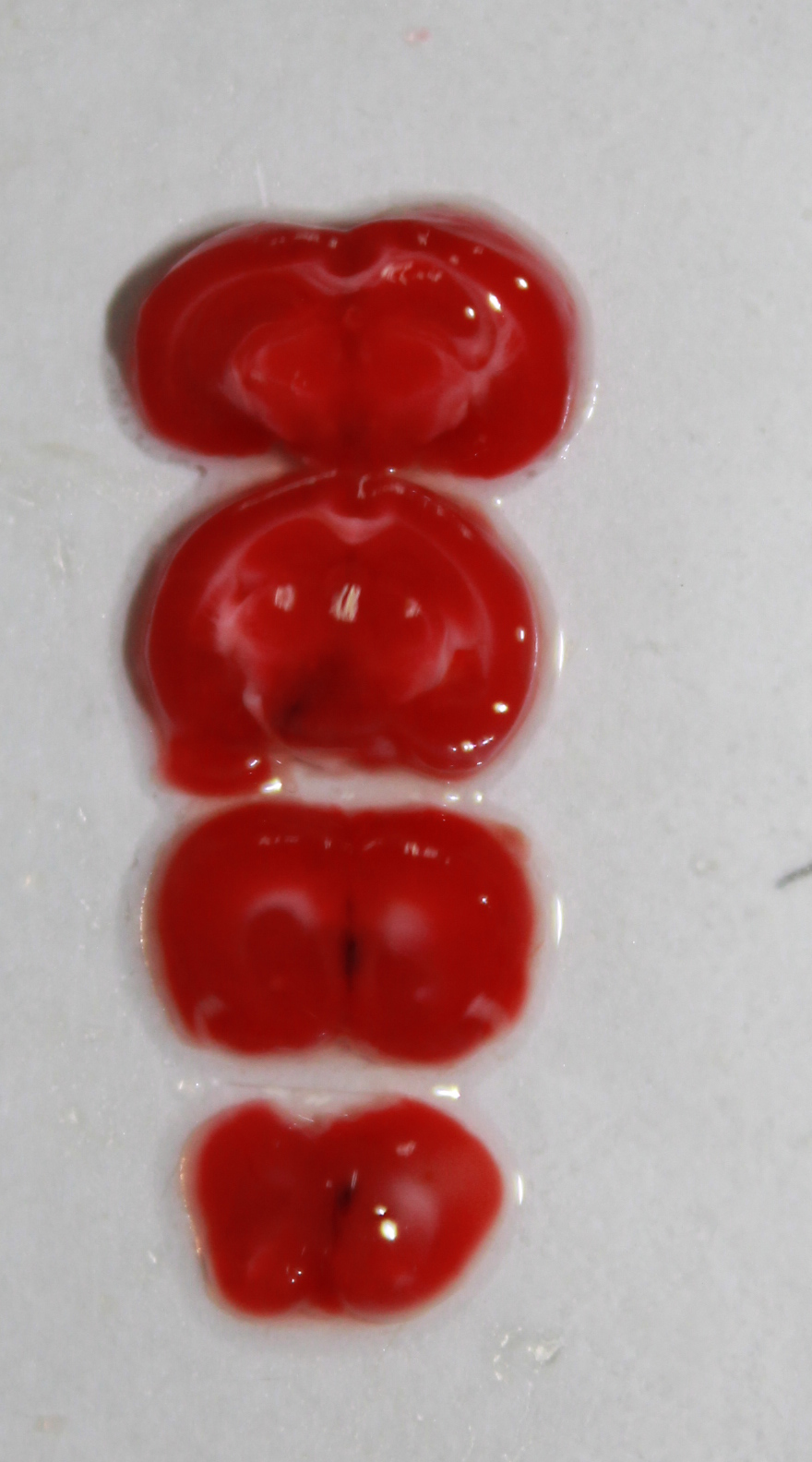

Supplement: Supplemental Information 1 [file peerj-11-14483-s001.zip › Original figures for cerebral infarction/ICT/ICT5.jpg]

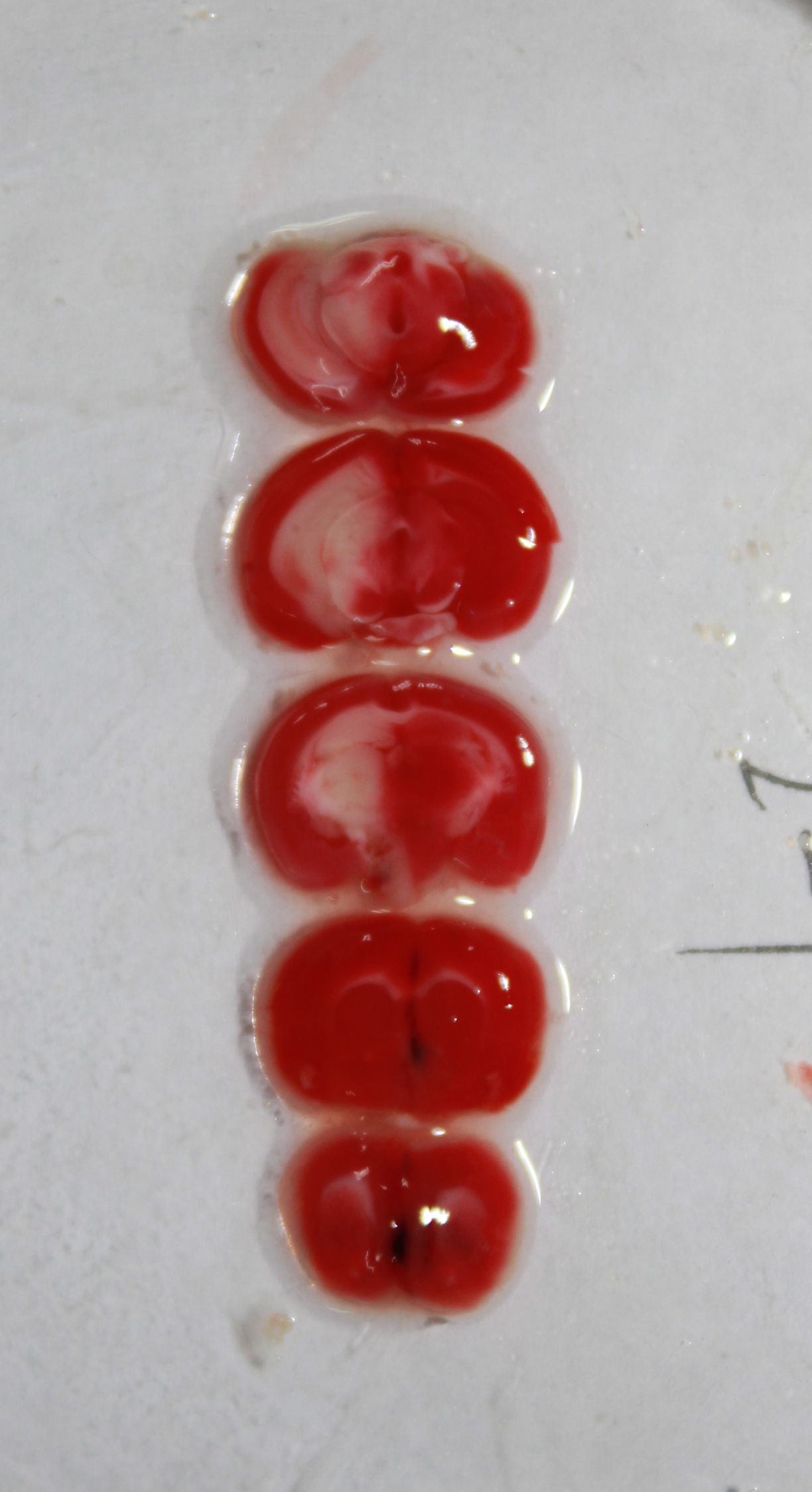

Supplement: Supplemental Information 1 [file peerj-11-14483-s001.zip › Original figures for cerebral infarction/ICT/ICT6.jpg]

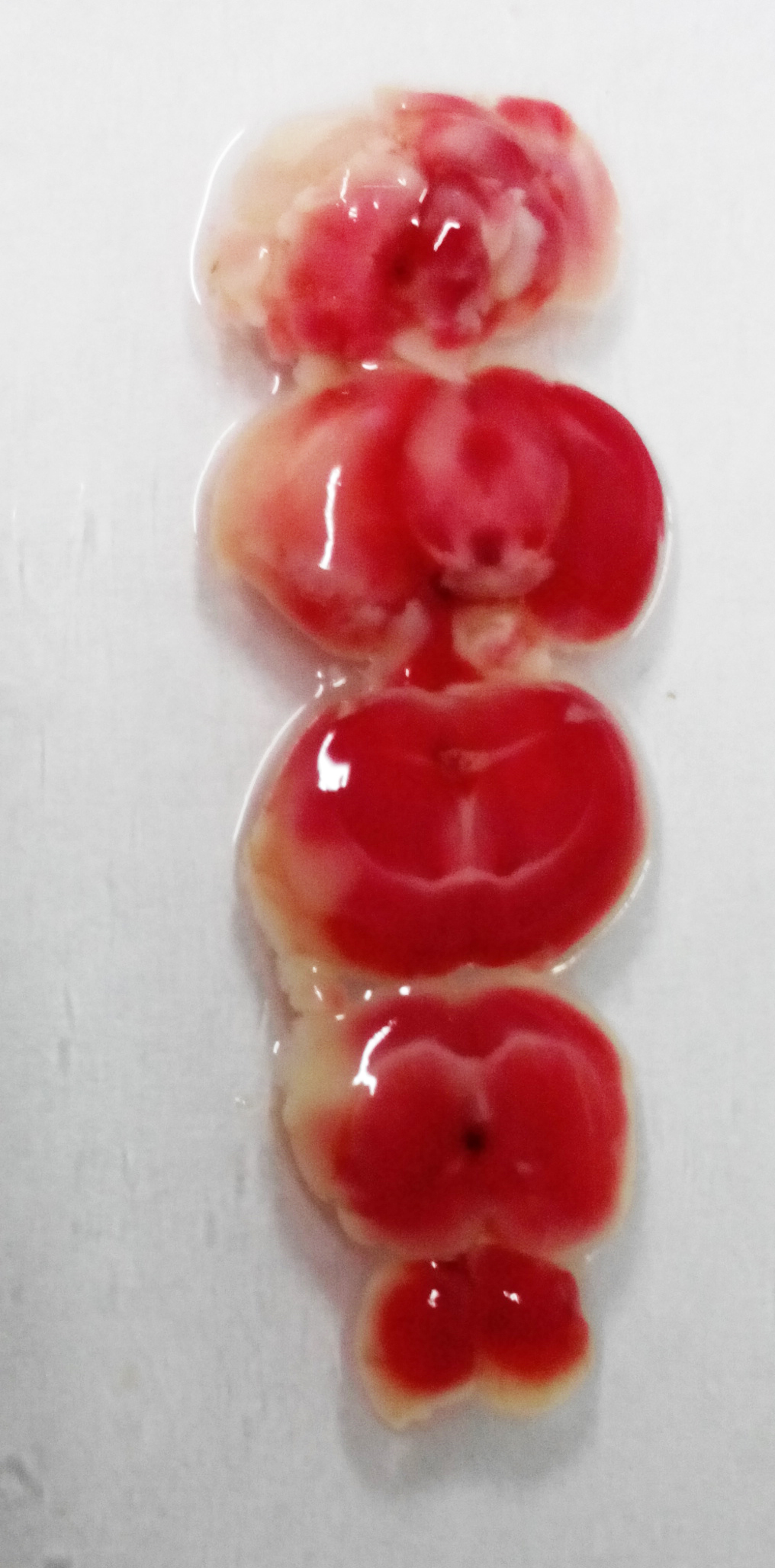

Supplement: Supplemental Information 1 [file peerj-11-14483-s001.zip › Original figures for cerebral infarction/ICT/ICT7.jpg]

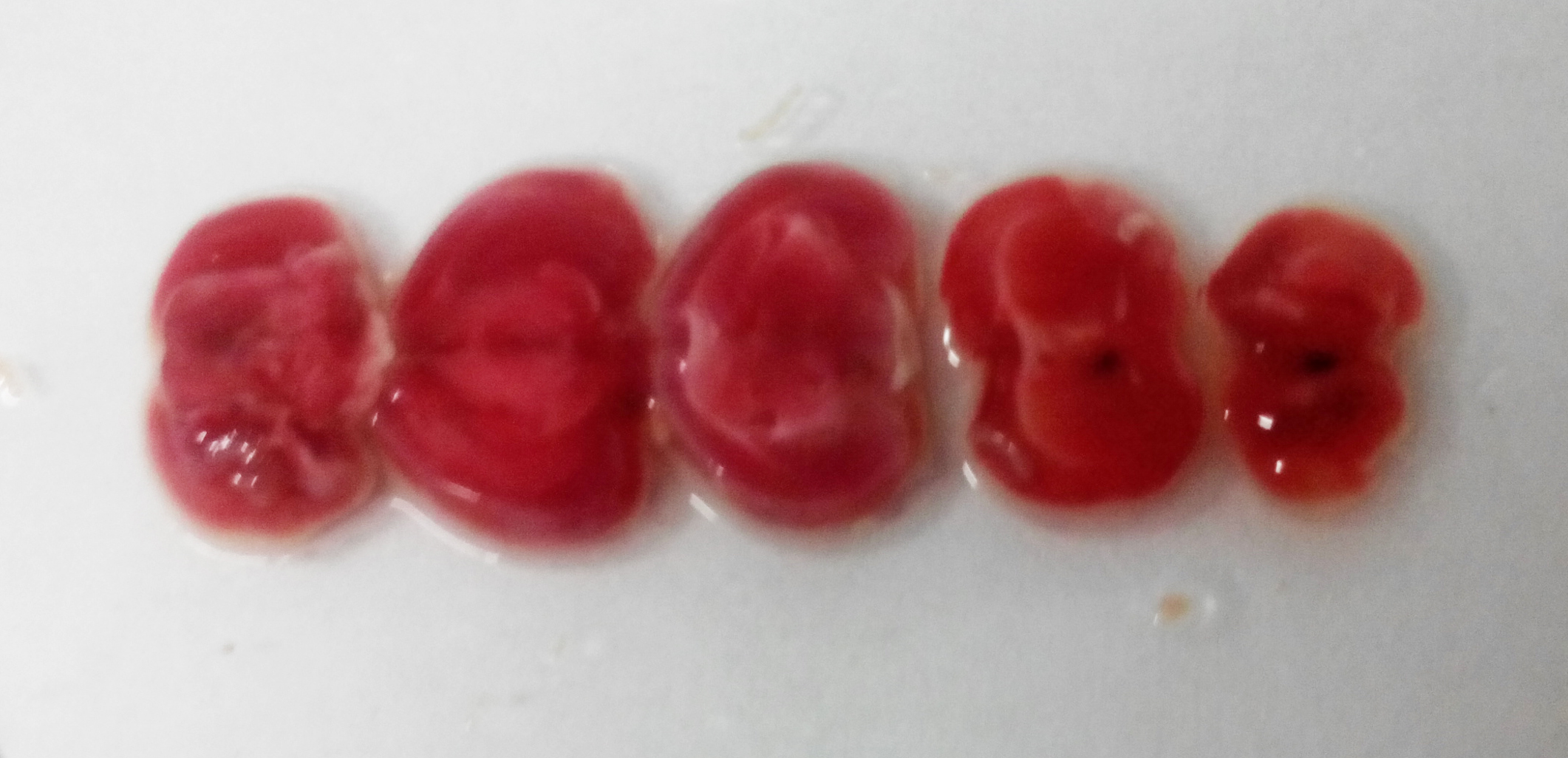

Supplement: Supplemental Information 1 [file peerj-11-14483-s001.zip › Original figures for cerebral infarction/ICT/ICT8.jpg]

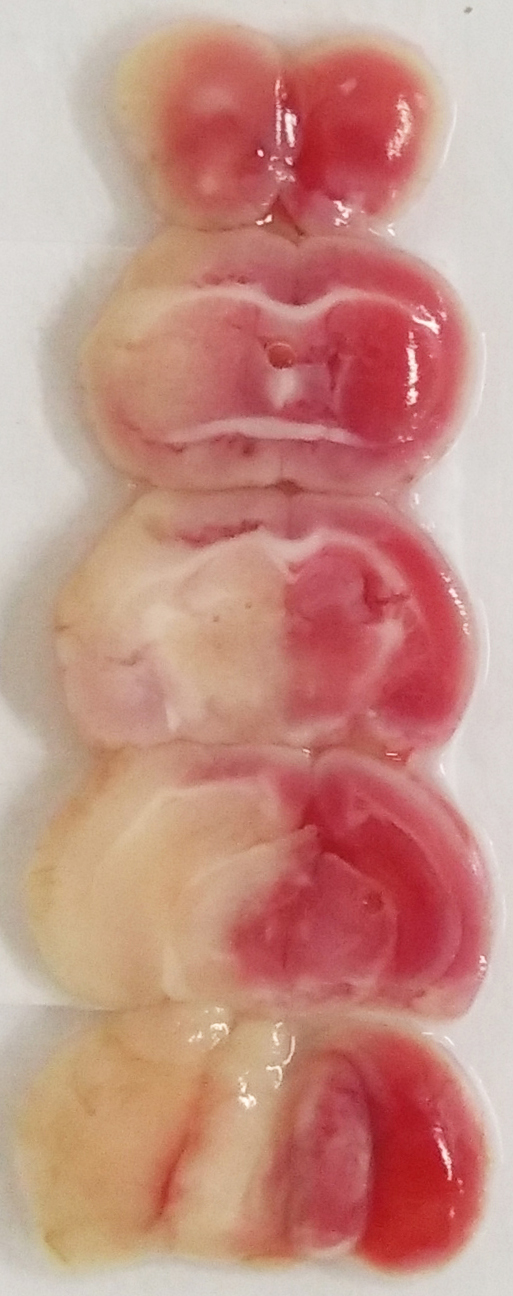

Supplement: Supplemental Information 1 [file peerj-11-14483-s001.zip › Original figures for cerebral infarction/Model/Model1.jpg]

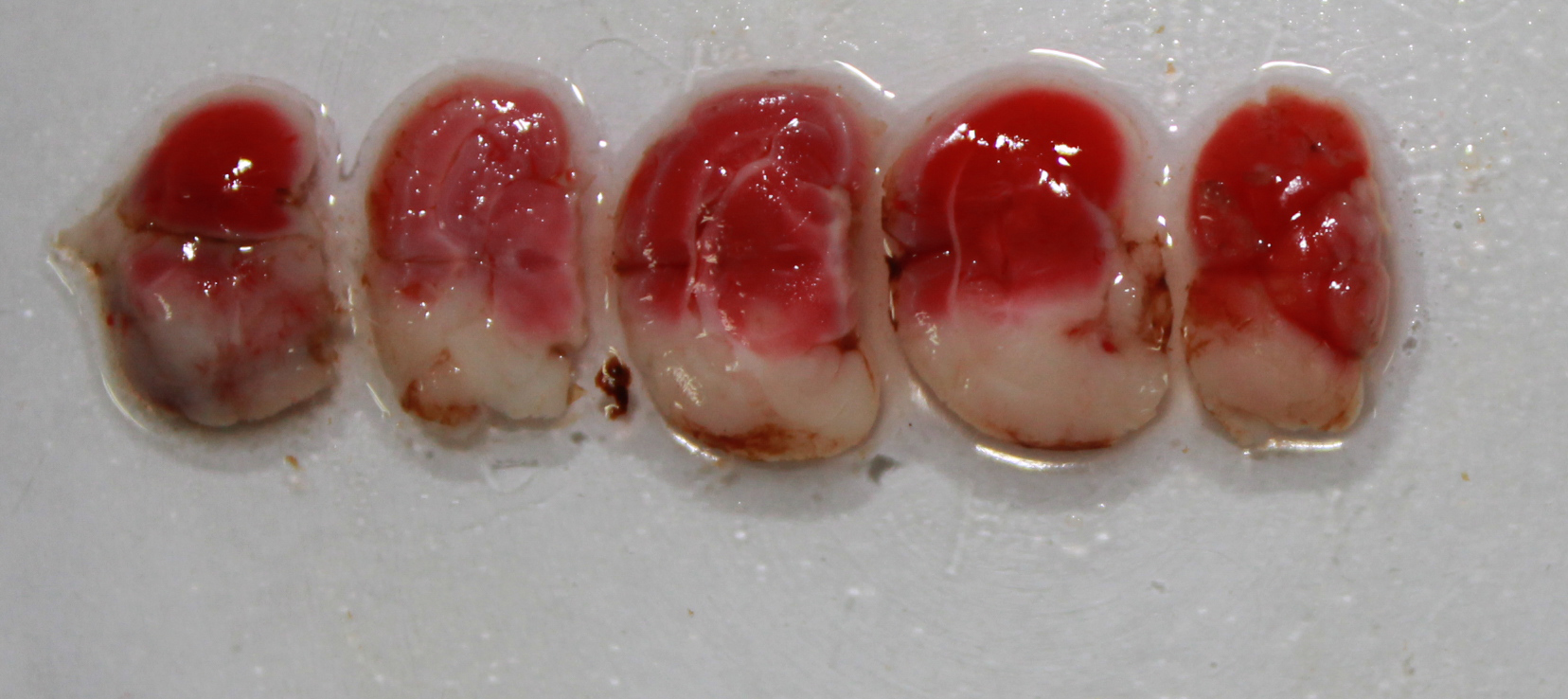

Supplement: Supplemental Information 1 [file peerj-11-14483-s001.zip › Original figures for cerebral infarction/Model/Model2.jpg]

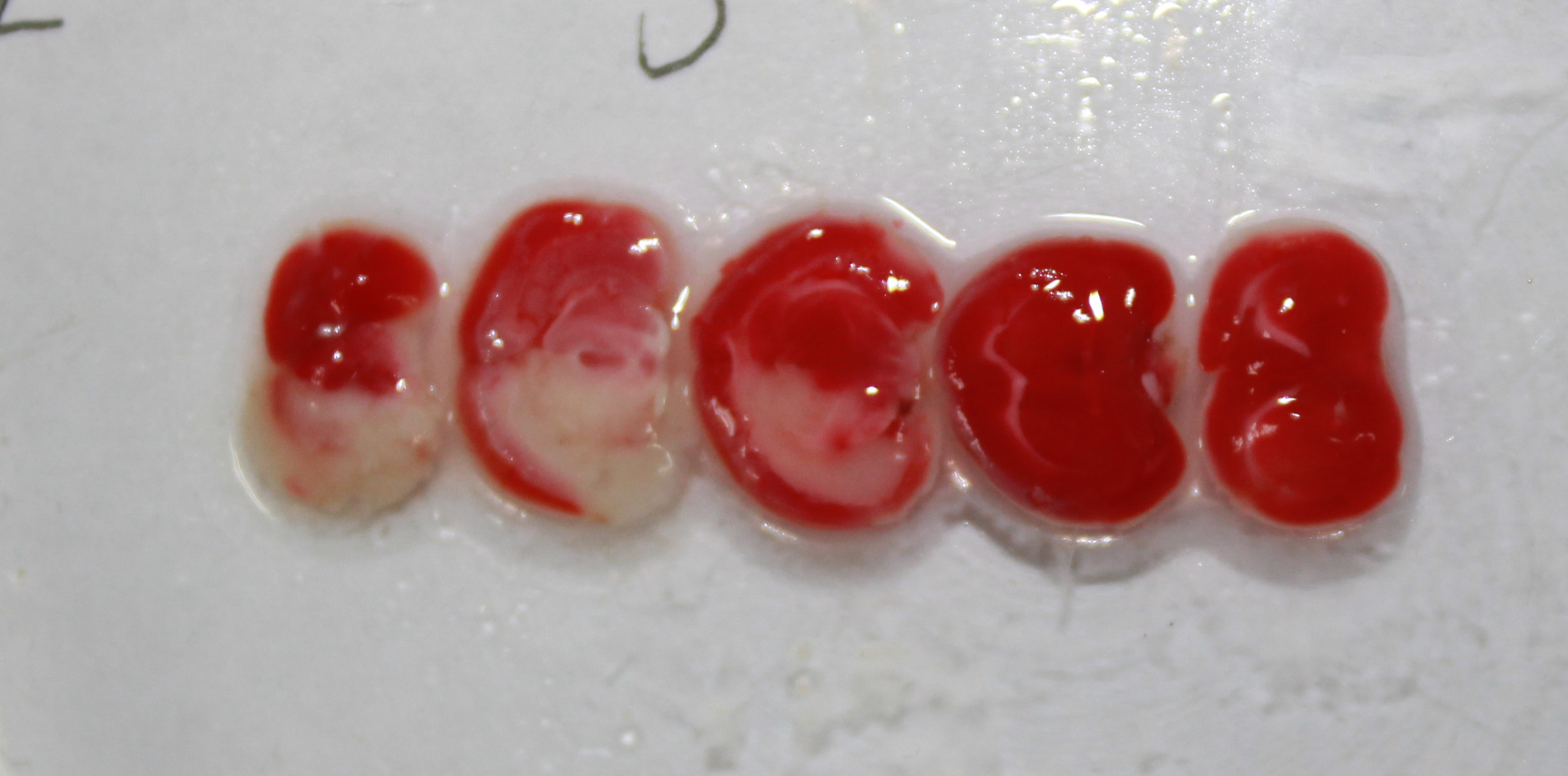

Supplement: Supplemental Information 1 [file peerj-11-14483-s001.zip › Original figures for cerebral infarction/Model/Model3.jpg]

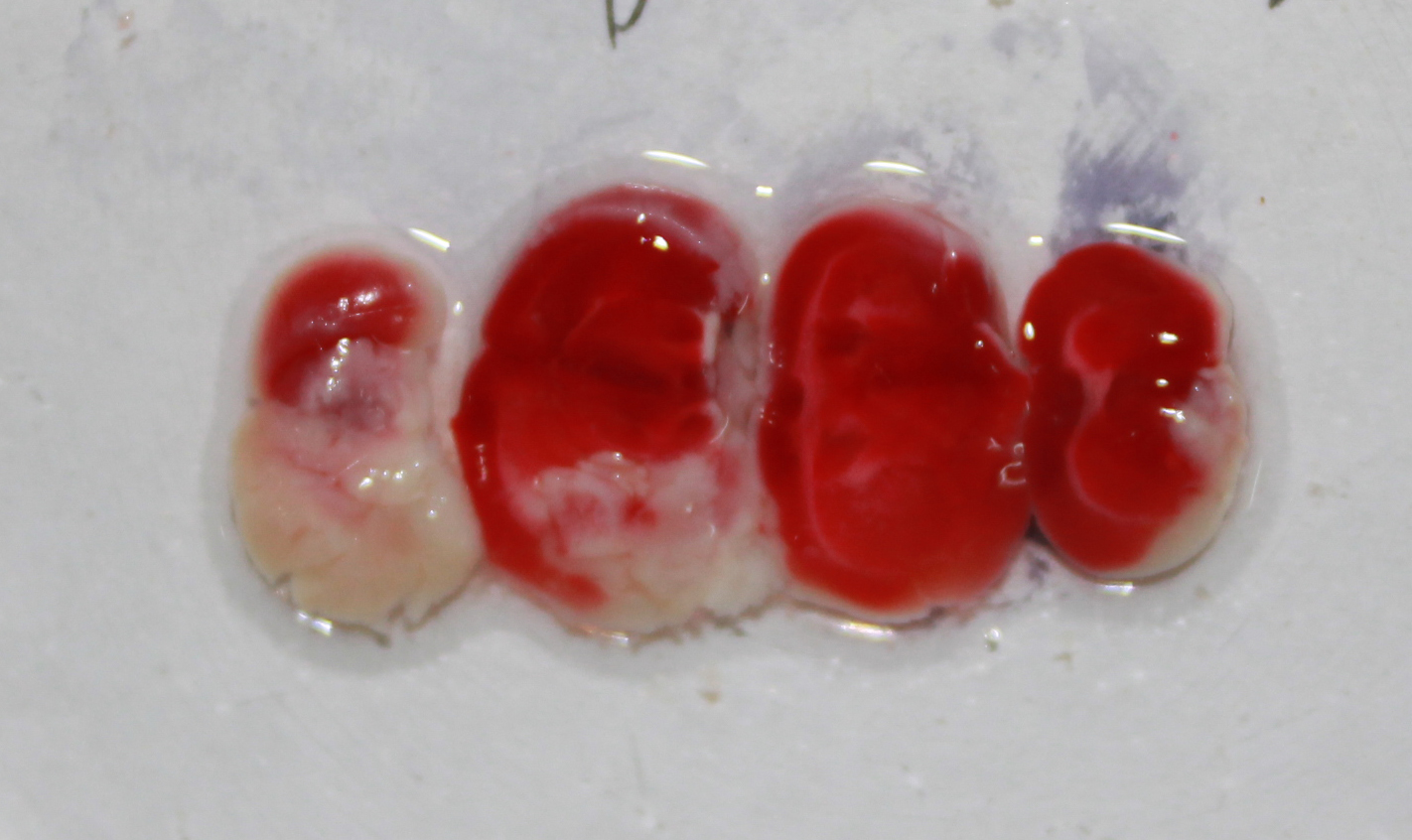

Supplement: Supplemental Information 1 [file peerj-11-14483-s001.zip › Original figures for cerebral infarction/Model/Model4.jpg]

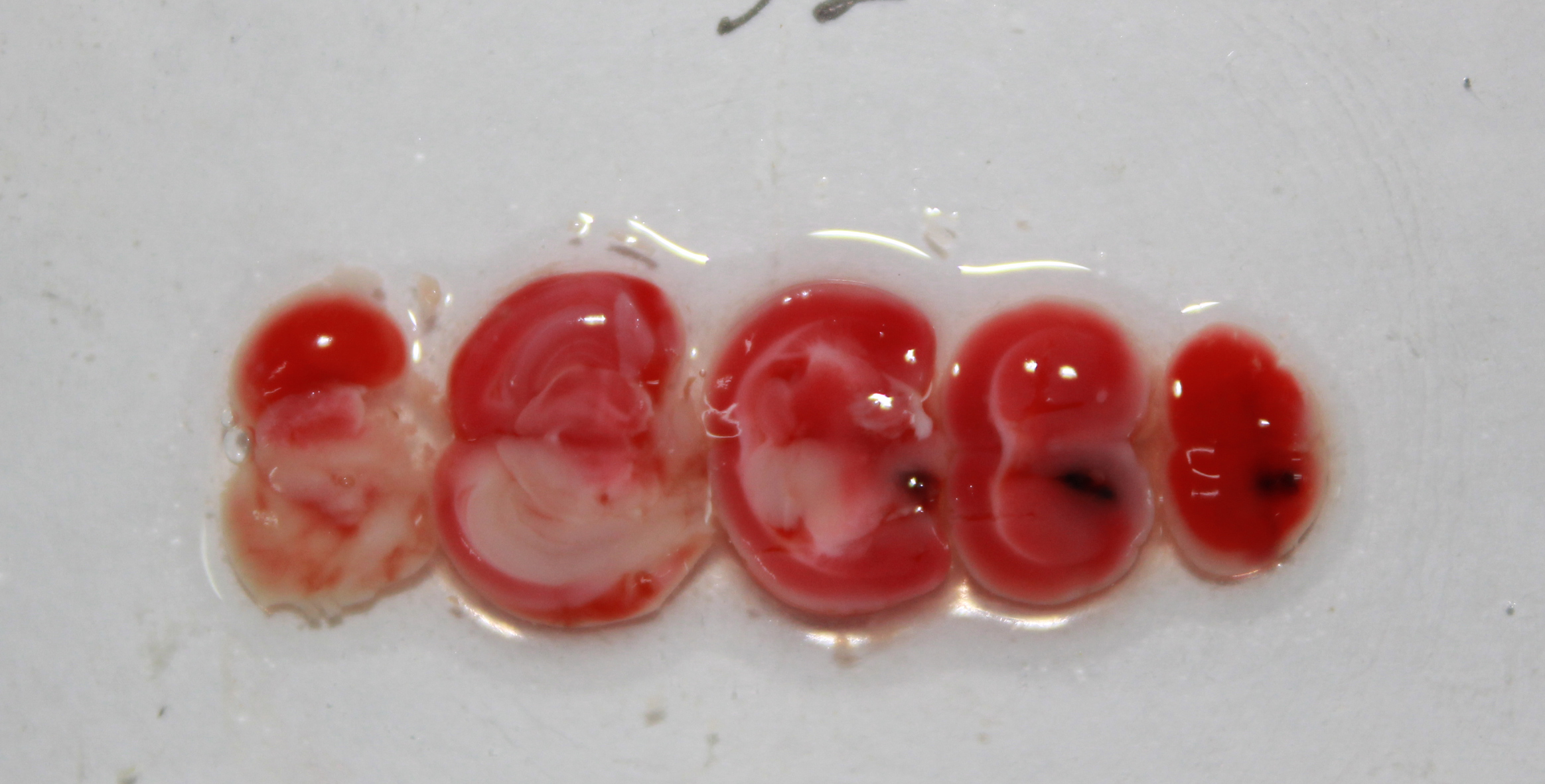

Supplement: Supplemental Information 1 [file peerj-11-14483-s001.zip › Original figures for cerebral infarction/Model/Model5.jpg]

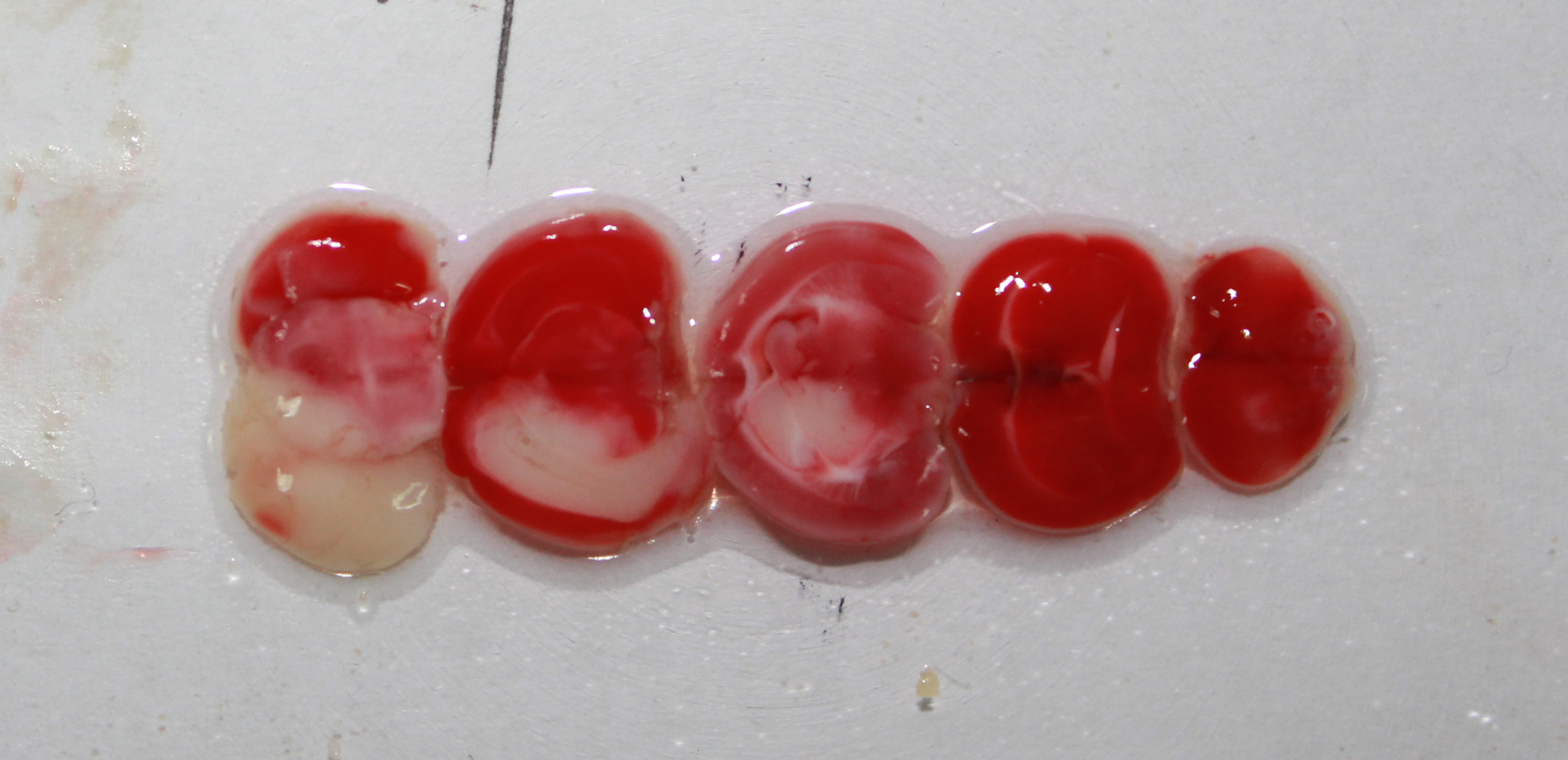

Supplement: Supplemental Information 1 [file peerj-11-14483-s001.zip › Original figures for cerebral infarction/Model/Model6.jpg]

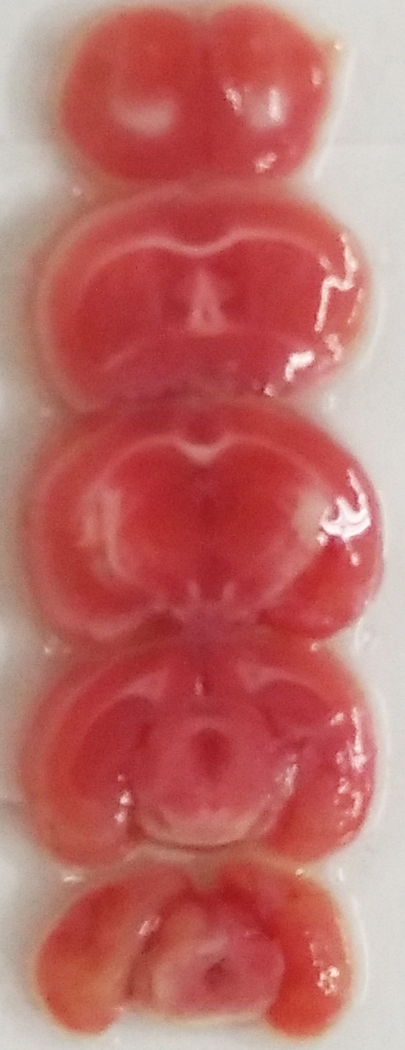

Supplement: Supplemental Information 1 [file peerj-11-14483-s001.zip › Original figures for cerebral infarction/Sham/Sham1.jpg]

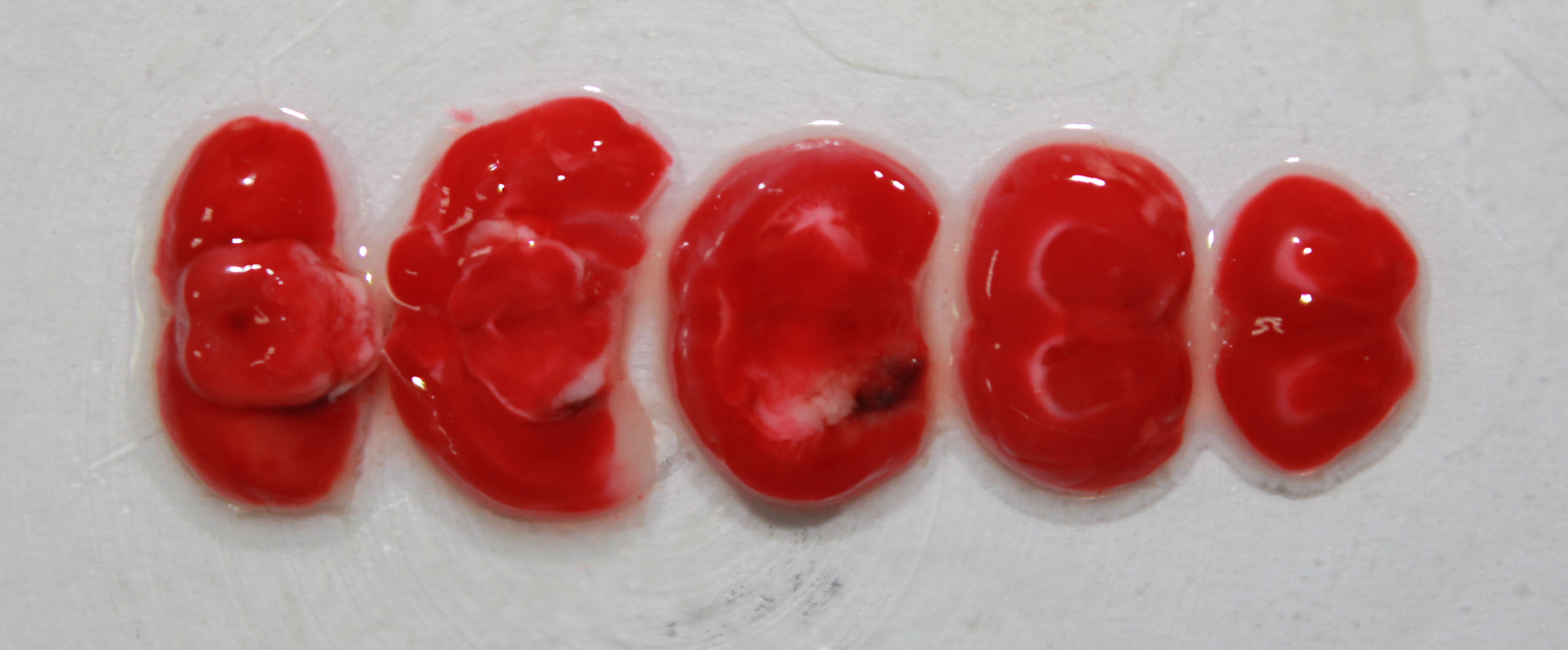

Supplement: Supplemental Information 1 [file peerj-11-14483-s001.zip › Original figures for cerebral infarction/Sham/Sham10.jpg]

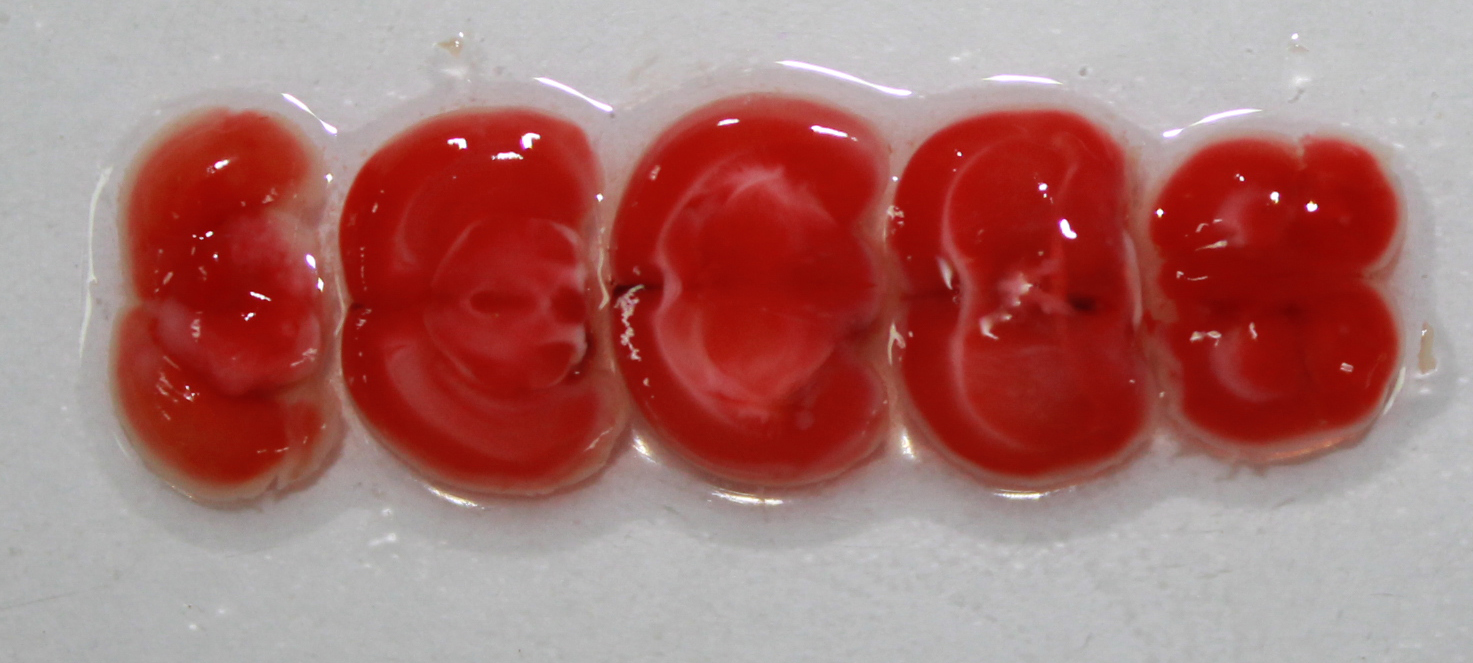

Supplement: Supplemental Information 1 [file peerj-11-14483-s001.zip › Original figures for cerebral infarction/Sham/Sham2.jpg]

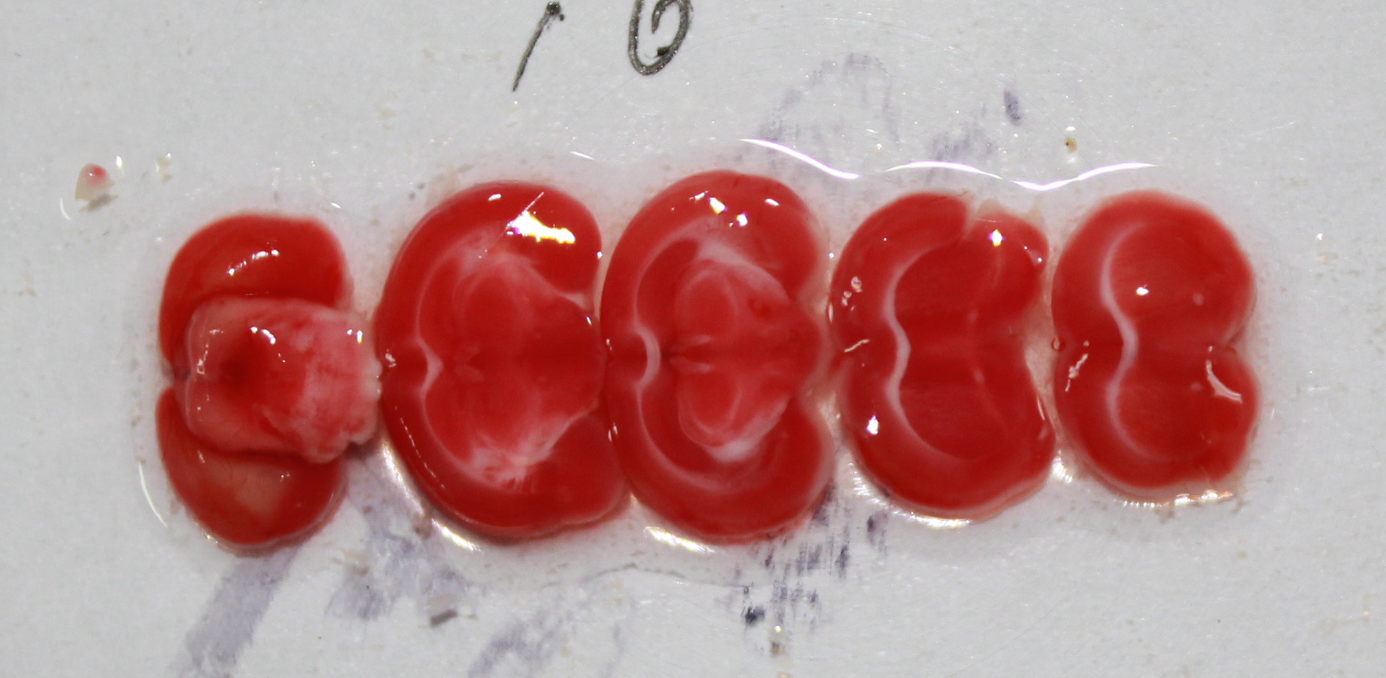

Supplement: Supplemental Information 1 [file peerj-11-14483-s001.zip › Original figures for cerebral infarction/Sham/Sham3.jpg]

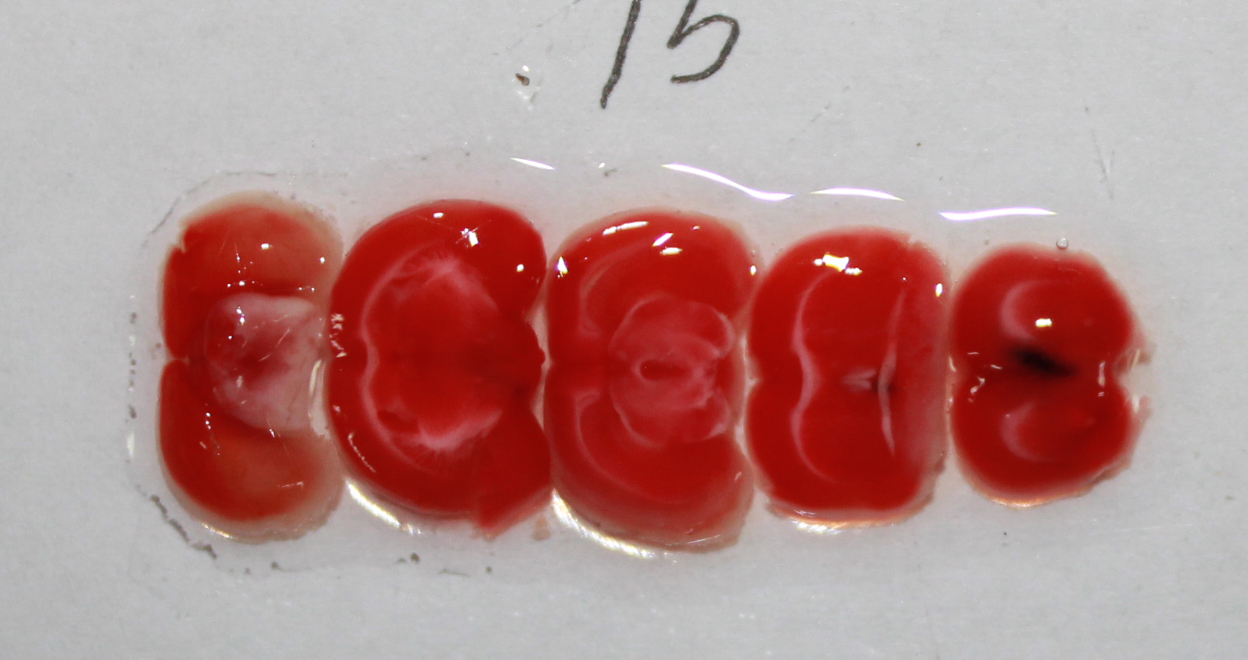

Supplement: Supplemental Information 1 [file peerj-11-14483-s001.zip › Original figures for cerebral infarction/Sham/Sham4.jpg]

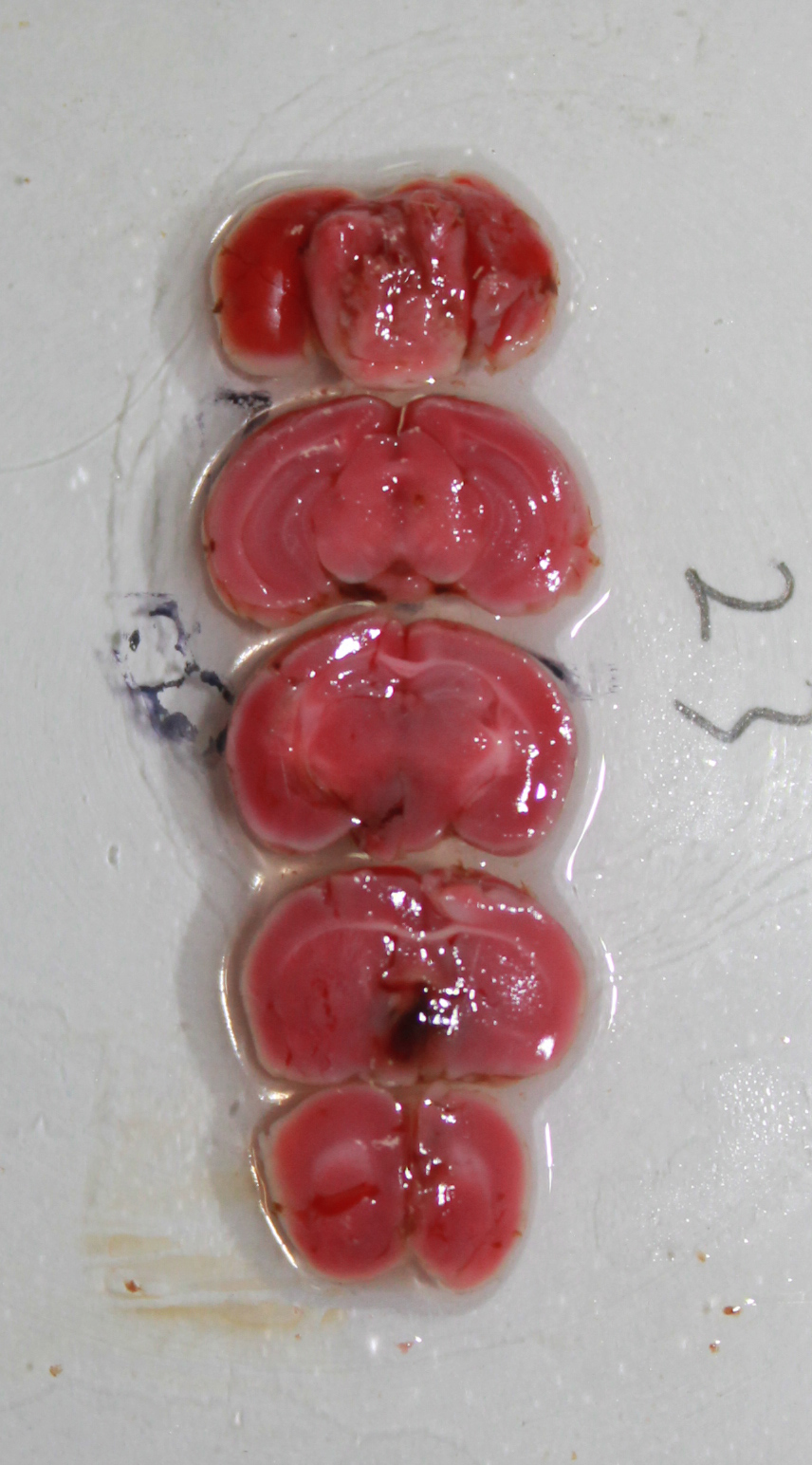

Supplement: Supplemental Information 1 [file peerj-11-14483-s001.zip › Original figures for cerebral infarction/Sham/Sham5.jpg]

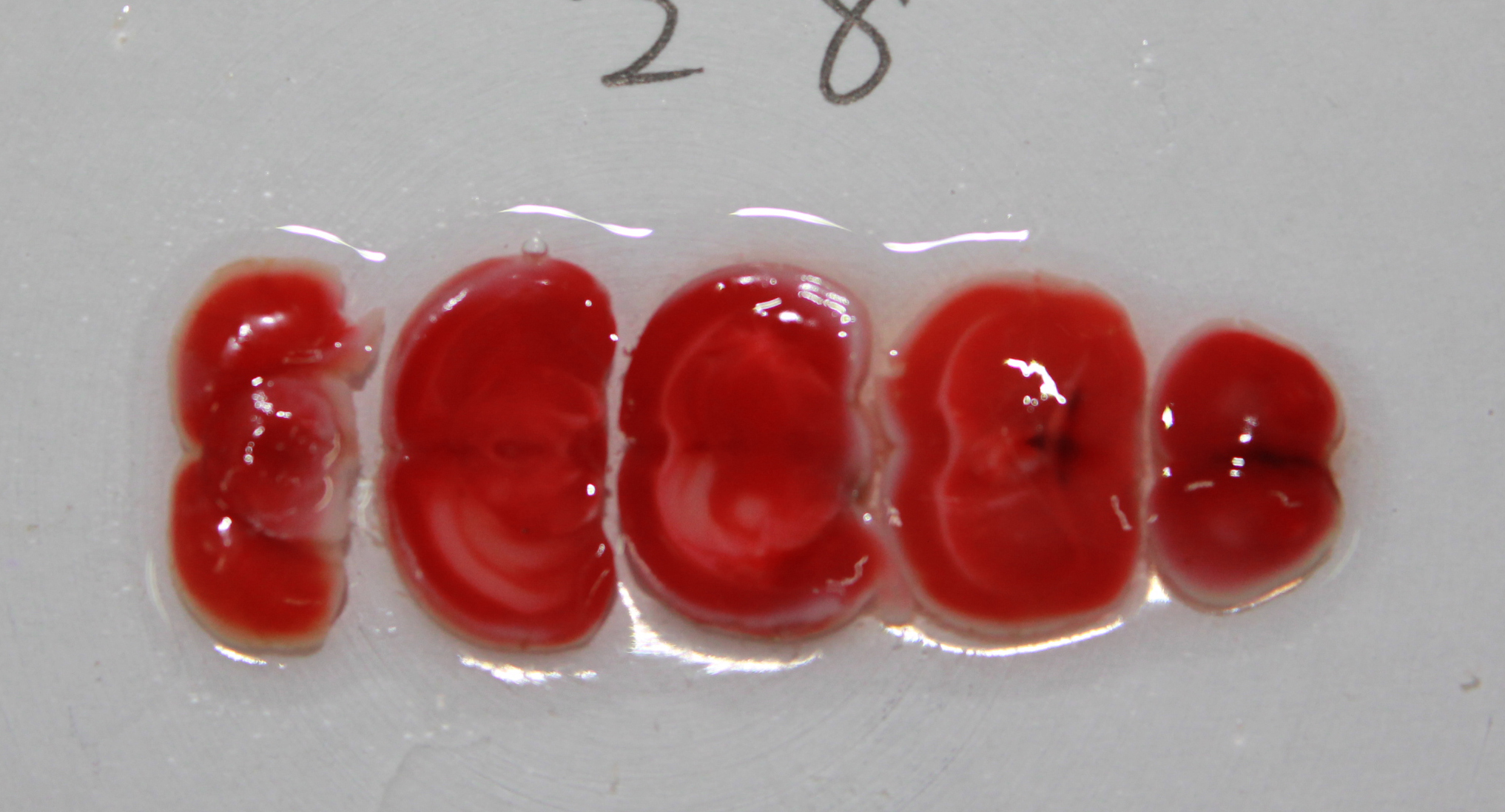

Supplement: Supplemental Information 1 [file peerj-11-14483-s001.zip › Original figures for cerebral infarction/Sham/Sham6.jpg]

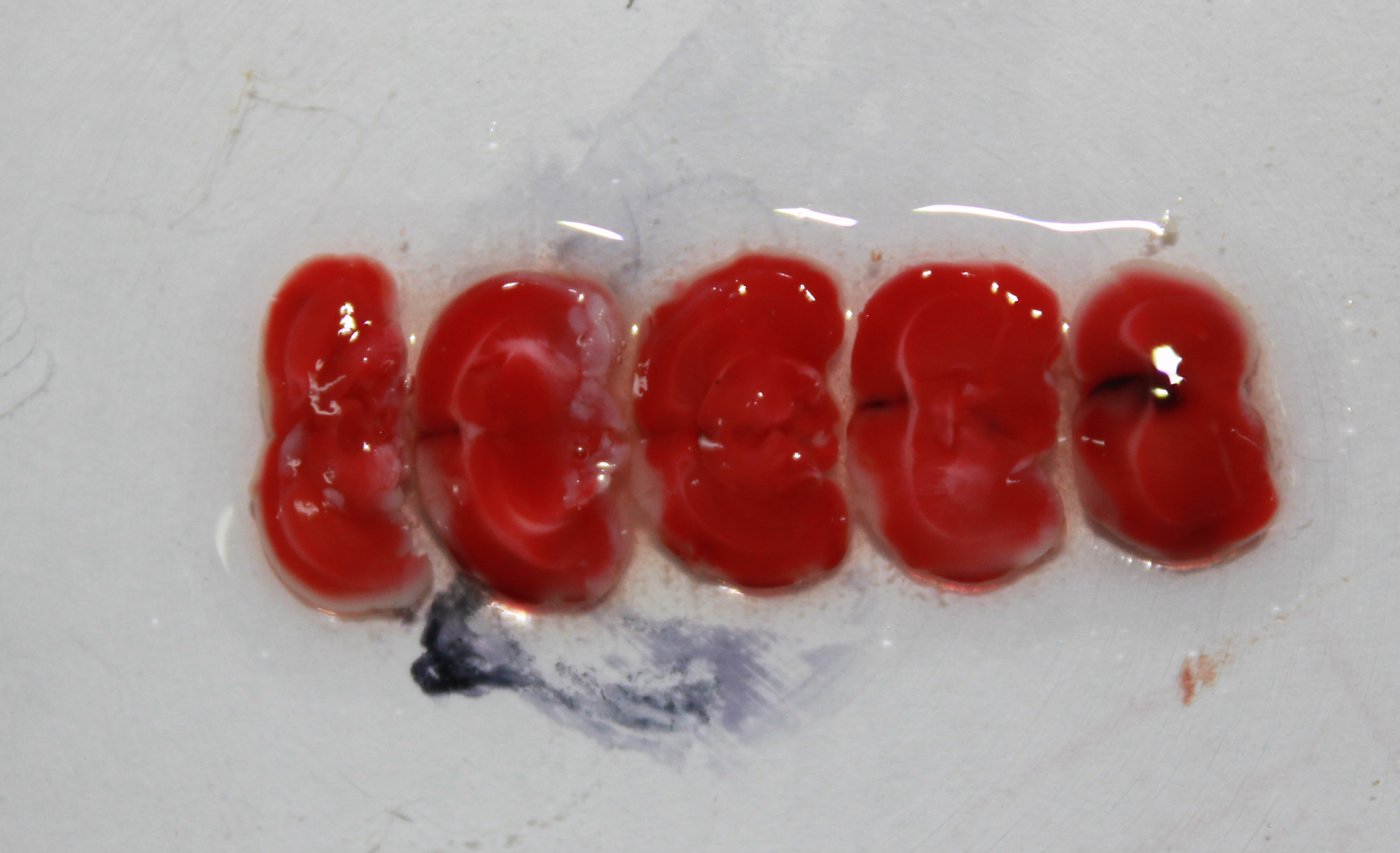

Supplement: Supplemental Information 1 [file peerj-11-14483-s001.zip › Original figures for cerebral infarction/Sham/Sham7.jpg]

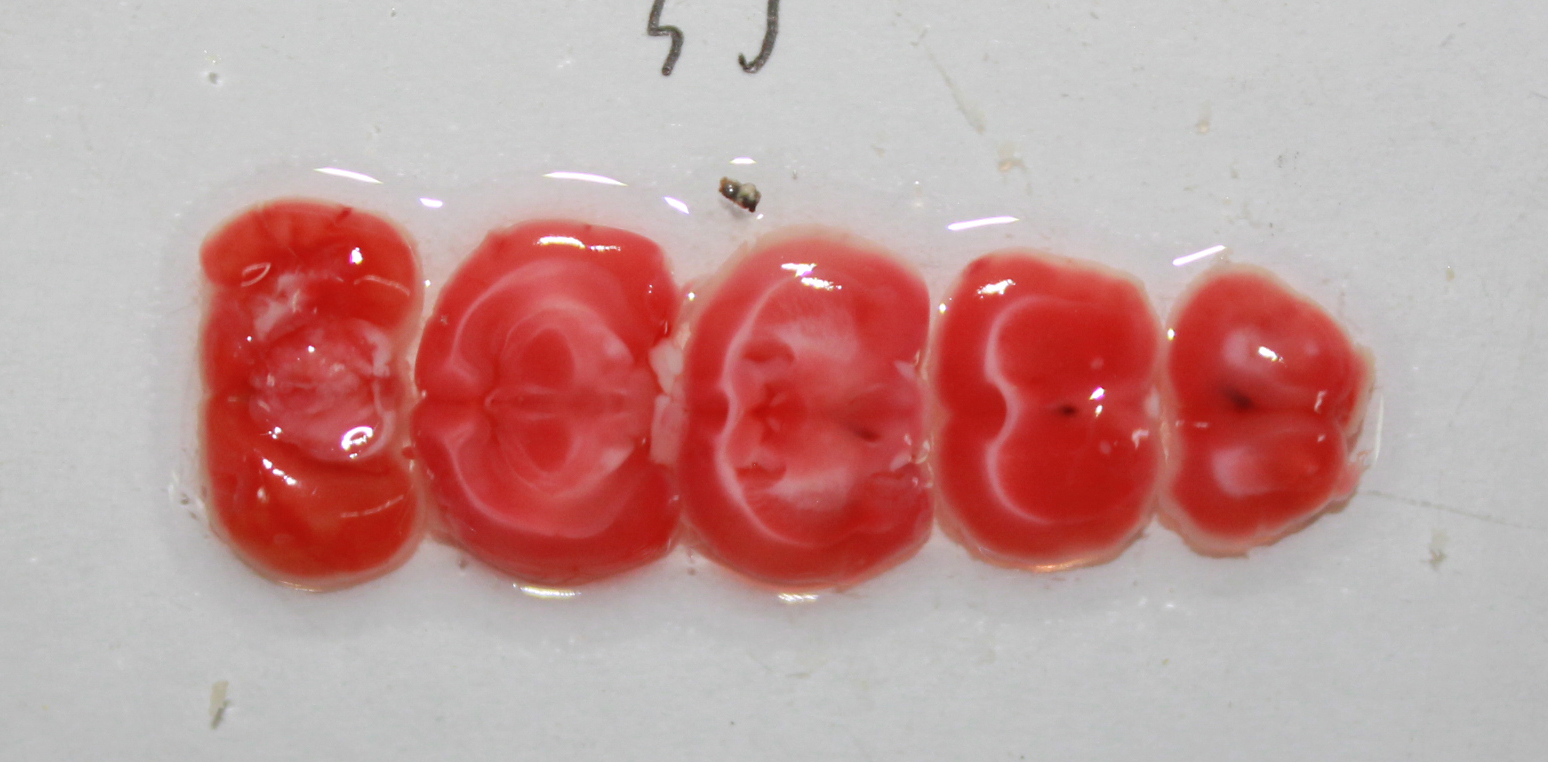

Supplement: Supplemental Information 1 [file peerj-11-14483-s001.zip › Original figures for cerebral infarction/Sham/Sham8.jpg]

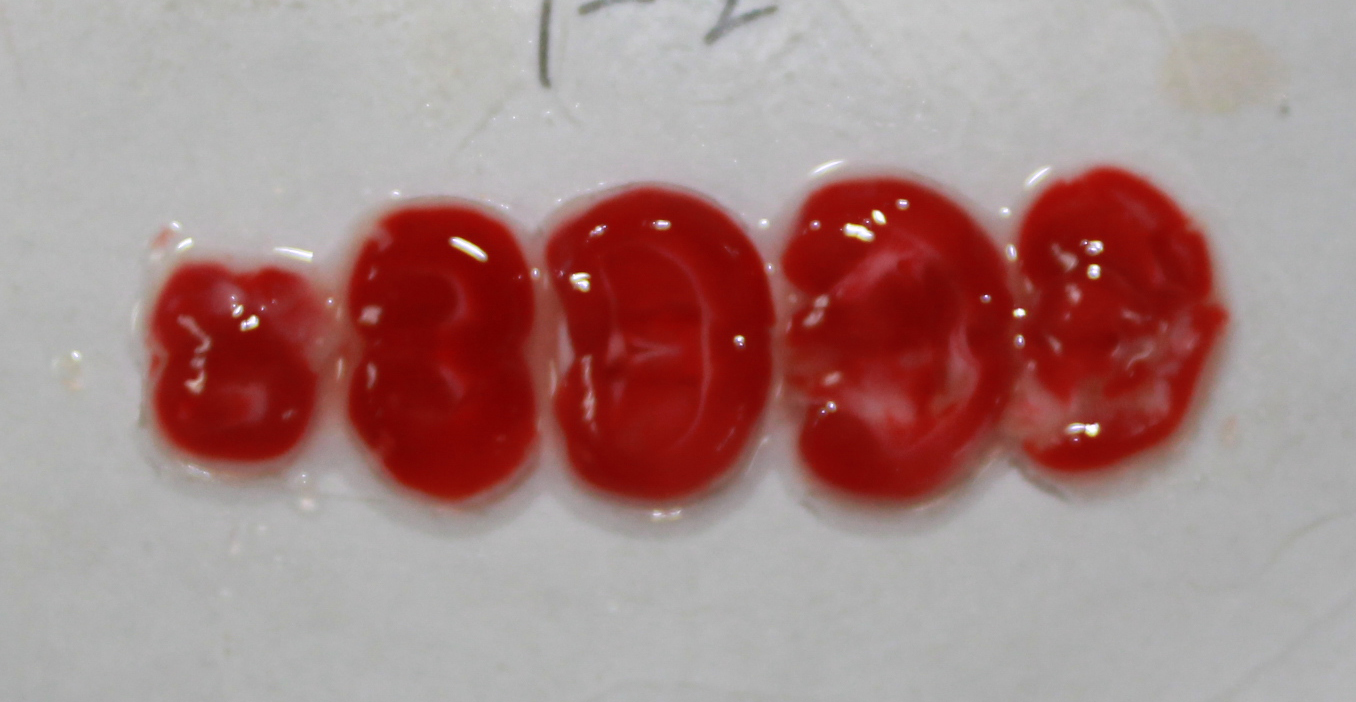

Supplement: Supplemental Information 1 [file peerj-11-14483-s001.zip › Original figures for cerebral infarction/Sham/Sham9.jpg]

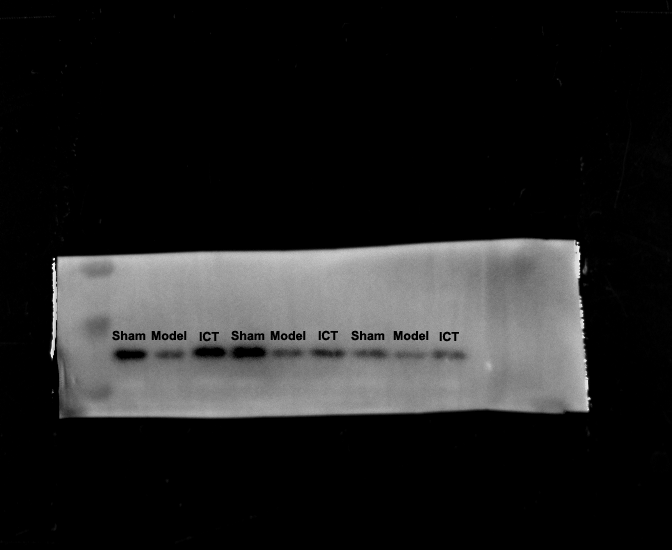

Supplement: Supplemental Information 4 [file peerj-11-14483-s004.zip › Original data for western blot/HO-1.tif]

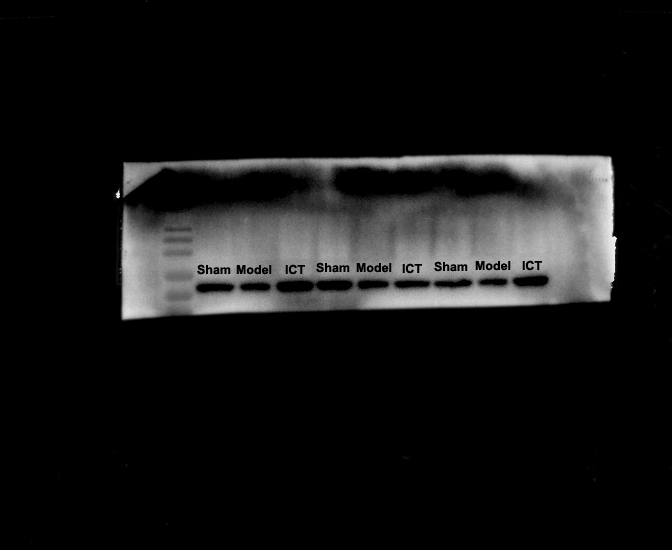

Supplement: Supplemental Information 4 [file peerj-11-14483-s004.zip › Original data for western blot/Nrf2.tif]

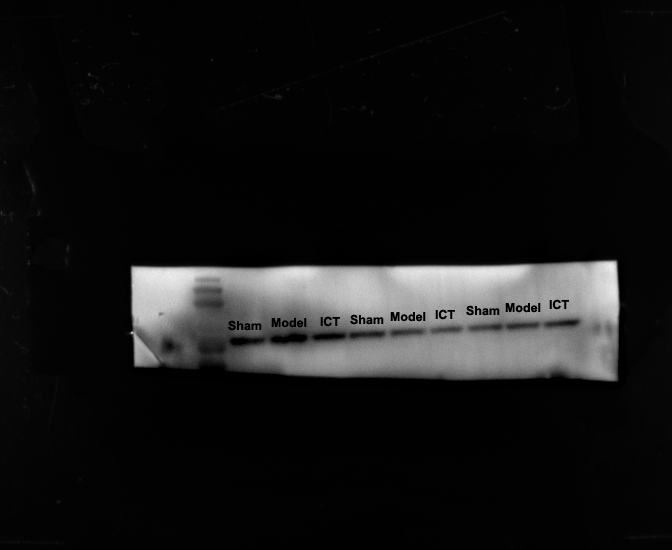

Supplement: Supplemental Information 4 [file peerj-11-14483-s004.zip › Original data for western blot/a-tubulin.tif]

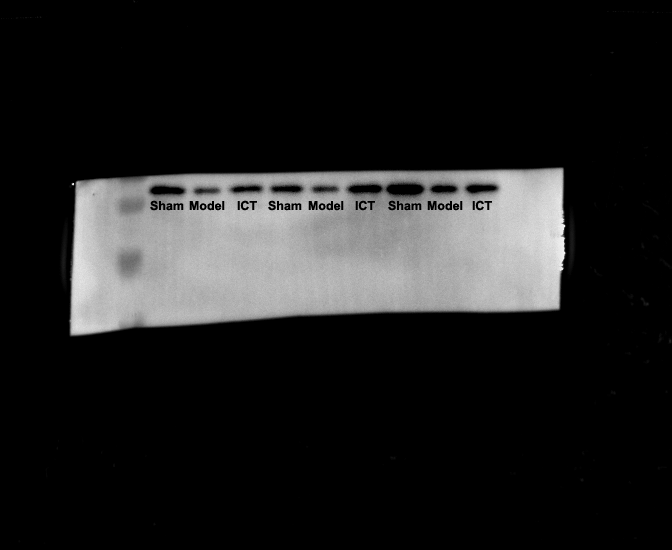

Supplement: Supplemental Information 4 [file peerj-11-14483-s004.zip › Original data for western blot/nqo-1.tif]
